# Simultaneous and High-Throughput Analytical Strategy of 30 Fluorinated Emerging Pollutants Using UHPLC-MS/MS in the Shrimp Aquaculture System

**DOI:** 10.3390/foods13203286

**Published:** 2024-10-16

**Authors:** Di Huang, Chengbin Liu, Huatian Zhou, Xianli Wang, Qicai Zhang, Xiaoyu Liu, Zhongsheng Deng, Danhe Wang, Yameng Li, Chunxia Yao, Weiguo Song, Qinxiong Rao

**Affiliations:** 1 The Institute of Agro-Food Standards and Testing Technology, Shanghai Academy of Agricultural Sciences, Shanghai 201403, China; hdsaas@126.com (D.H.); liuchengbin@saas.sh.cn (C.L.); 15861639745@163.com (H.Z.); wangxianli@saas.sh.cn (X.W.); qicaizhang@126.com (Q.Z.); 16678050954@163.com (X.L.); zhongshengdeng668@163.com (Z.D.); wdh@saas.sh.cn (D.W.); liyameng@saas.sh.cn (Y.L.); chunxiayao2007@saas.sh.cn (C.Y.); qinxiongrao@saas.sh.cn (Q.R.); 2 Key Laboratory of Food Quality Safety and Nutrition (Co-Construction by Ministry and Province), Ministry of Agriculture and Rural Affairs, Shanghai 201403, China; 3 Shanghai Co-Elite Agri-Food Testing Technical Service Co., Ltd., Shanghai 201403, China; 4 School of Health Science and Engineering, University of Shanghai for Science & Technology, Shanghai 100049, China

**Keywords:** shrimp aquaculture system, fluorinated emerging pollutants, simultaneous analysis, UHPLC-MS/MS

## Abstract

This study established novel and high-throughput strategies for the simultaneous analysis of 30 fluorinated emerging pollutants in different matrices from the shrimp aquaculture system in eastern China using UHPLC-MS/MS. The parameters of SPE for analysis of water samples and of QuEChERS methods for sediment and shrimp samples were optimized to allow the simultaneous detection and quantitation of 17 per- and polyfluoroalkyl substances (PFASs) and 13 fluoroquinolones (FQs). Under the optimal conditions, the limits of detection of 30 pollutants for water, sediment, and shrimp samples were 0.01–0.30 ng/L, 0.01–0.22 μg/kg, and 0.01–0.23 μg/kg, respectively, while the limits of quantification were 0.04–1.00 ng/L, 0.03–0.73 μg/kg, and 0.03–0.76 μg/kg, with satisfactory recoveries and intra-day precision. The developed methods were successfully applied to the analysis of multiple samples collected from aquaculture ponds in eastern China. PFASs were detected in all samples with concentration ranges of 0.18–0.77 μg/L in water, 0.13–1.41 μg/kg (dry weight) in sediment, and 0.09–0.96 μg/kg (wet weight) in shrimp, respectively. Only two FQs, ciprofloxacin and enrofloxacin, were found in the sediment and shrimp. In general, this study provides valuable insights into the prevalence of fluorinated emerging contaminants, assisting in the monitoring and control of emerging contaminants in aquatic foods.

## 1. Introduction

Shrimp farming represents one of the most economically productive aquaculture industries globally, particularly within the Asia–Pacific region, with annual export revenues reaching billions of dollars. The South American white shrimp exhibits several advantageous characteristics, including a relatively low content of fat, a high protein concentration, and a good flavor profile. Consequently, it represents the dominant species in global shrimp farming, accounting for approximately 80% of the world’s total production of farmed shrimp [1,2]. In aquaculture systems, ponds have the potential to become sinks for pollutants as a result of the combined influence of environmental and anthropogenic factors [3,4,5]. Previous studies have focused on the analysis of pesticides and veterinary drugs in aquatic organisms and aquaculture environment [6,7,8,9,10]. Due to the increasing concern about emerging pollutants in recent years, current research has focused on the impact of emerging pollutants on aquatic organisms [11,12,13,14].

Emerging pollutants in the environment have been of widespread concern in recent years. As two main types of emerging pollutants, persistent organic pollutants (POPs), represented by per- and polyfluoroalkyl substances (PFASs), and antibiotics, such as fluoroquinolones (FQs), have raised national and international attention [15,16,17,18]. As fluorine is the most electronegative element, fluorinated compounds tend to be stable and are widely used in industry, agriculture, and pharmaceuticals. PFASs are a representative group of fluorinated emerging pollutants that are widespread in the environment [19,20,21,22,23]. The production, use, and disposal have resulted in direct or indirect releases of PFASs into the environment, which can accumulate in organisms and humans via food chains, posing a serious threat to health [24,25]. Therefore, several PFASs with high risk have been listed and subject to control in the Stockholm Convention on Persistent Organic Pollutants [26]. As another typical fluorinated emerging pollutants, FQs have been frequently found in wastewater, biological matrices, soils, and sediments and have become one of the major antibiotic residues in aquatic systems [27,28,29,30,31]. Moreover, FQs can lead to the antibiotic resistance in bacteria, resulting in negative impacts on the ecosystem and human health [32,33]. Currently, the maximum residue levels of 10 FQs, such as dafloxacin and enrofloxacin, have been restricted in national food safety standards [34].

The development of general methods that allow the simultaneous determination of multiple classes of compounds has been the trend in recent years for the analysis of contaminants in the environment [35,36,37,38,39]. It is notable that there are differences in the pretreatment methods for contaminants in environmental and biological matrices. Solid phase extraction (SPE) is a commonly used technique for the pretreatment of water samples, which enables the selective concentration of targeted analytes from aqueous solutions via solid adsorbents [40,41,42]. In the case of contaminants in solid matrices, such as sediment or organisms, the QuEChERS (Quick, Easy, Cheap, Effective, Robust, and Safe) technique is frequently employed for the purpose of pre-treatment [43,44]. Other analytical techniques have also been applied for the pretreatment of target analytes. For example, single-drop microextraction was used for the pre-concentration of FQs in water and urine samples [45,46], and dispersive liquid–liquid microextraction using nanoparticles was applied for the pretreatment of PFASs in aqueous samples [47,48]. However, these techniques are not as widely used as SPE and QuEChERS methods due to their limitations such as high operational requirements, and time consuming and complex steps. Following purification and concentration, the samples are subjected to analysis using analytical instruments such as chromatography or mass spectrometry. Liquid chromatography–tandem mass spectrometry (LC-MS/MS) is a widely used instrument for the analysis of PFASs and FQs in contemporary studies [37,49,50]. However, in previously reported analytical techniques, PFASs and FQs are typically pretreated independently under different pH conditions and the quantitative detection methods are primarily focused on a single type of compounds [51,52]. There was still insufficiency on the high-throughput of different kinds of emerging pollutants in shrimps and their habitats from aquaculture systems, especially like the fluorinated emerging pollutants with high persistence and severe threat.

The objective of this study was to develop simultaneous and high-throughput analytical methods for two classes of fluorinated emerging pollutants (17 PFASs and 13 FQs) in the shrimp aquaculture system. The parameters of chromatographic conditions, extraction, pretreatment procedures, and pretreatment processes which can affect the analytical efficiency were systematically optimized. The developed methods were then applied to the simultaneous analysis of different kinds of 30 fluorinated emerging pollutants in samples from shrimp aquaculture ponds in eastern China, in order to reveal the contamination of the fluorinated emerging pollutants in the shrimp aquaculture system. The findings of this study can assist in the monitoring and control of emerging pollutants in the aquaculture system. The research can also provide scientific data for understanding the migration, transformation, and bioaccumulation processes of fluorinated emerging pollutants and assess the environmental risk of contaminants in aquaculture systems.

## 2. Materials and Methods

### 2.1. Materials and Chemicals

Methanol, concentrated sulfuric acid (H_2_SO_4_, 98%), disodium ethylene diamine tetraacetate (Na_2_EDTA) of chromatographic grade and anhydrous magnesium sulphate (MgSO_4_), sodium chloride (NaCl), anhydrous sodium sulphate (Na_2_SO_4_) of analytical grade were purchased from Sinopharm Chemical Reagent Co. (Shanghai, China). Acetonitrile, formic acid of chromatographic grade and graphitized carbon black (GCB, 120–400 mesh) were procured from Anpel Laboratory Technologies Inc. (Shanghai, China). Ammonium acetate (chromatographic pure grade) and carboxylated multi-walled carbon nanotubes (MWCNTs, purity > 95%, inner diameter 3–5 nm, outer diameter 8–15 nm, length ~50 μm) were bought from Shanghai Aladdin Biochemical Technology Co. (Shanghai, China). MWCNT solid phase extraction columns were prepared by packing 500 mg of MWCNTs into empty SPE column tubes. HLB solid phase extraction columns (500 mg/6 mL) were purchased from Waters Corp. (Milford, MA, USA). C_18_ SPE columns (500 mg/6 mL) were bought from Shanghai Taopusai Biotechnology Co. (Shanghai, China). Ethylenediamine-N-propylsilane silica gel (primary secondary amine, PSA, 74–150 μm) was purchased from Guangzhou Bright Chemical Co. (Guangzhou, China).

Fluoroquinolone standards (Quinolone Mix-15, 100 μg/mL) and three internal standards (Norfloxacin-D_5_, Ciprofloxacin-D_8_, Enrofloxacin-D_5_, 100 μg/mL) were purchased from BePure (Beijing, China). The standards of PFASs (EPA Method 537.1 Mixtures, 2 μg/mL) were purchased from Cambridge Isotope Laboratories, Inc. (Andover, MA, USA). The internal standards of PFASs (MPFAC-MXA, 2 μg/mL) were purchased from Wellington Laboratories (Guelph, ON, Canada).

### 2.2. Sample Collection

A series of samples, including 16 water samples, 13 sediment samples, and 19 shrimp samples, were collected from shrimp aquaculture ponds during August and September 2023 in Shanghai. Further details about the samples can be found in the Supplementary Materials (Table S1), including the sample number, date of sampling, coordinates of sampling sites, and sample types. The water sample, with a volume of 1 L, was collected from each sampling site using a sterile water sampling bag. A stainless steel substrate sampler was used to collect sediment samples from the bottom of each site at a depth of 0 to 10 cm according to a standardized procedure, and the collected samples were then sealed in a clean polyethylene bag for storage. To ensure the representativeness of the samples, healthy shrimp were randomly collected from each pond. All samples were sent to the laboratory within four hours and stored at −18 °C. The sediment samples were subjected to freeze-drying prior to preparation for subsequent analysis.

### 2.3. Standard Solution

A stock solution of 2 μg/mL of FQs was prepared by taking 20 μL of the standard solution (100 μg/mL) and diluting it in 980 μL of methanol. The mixed standards of 17 PFASs and 13 FQs at a concentration of 200 μg/L were prepared by adding 100 μL of PFASs standard solution (2 μg/mL) and 100 μL of the stock solution of FQs (2 μg/mL) to 800 μL of methanol.

### 2.4. Sample Pre-Treatment

In the present analytical strategy, we optimized the SPE columns, elution solvents, and volumes and pH of water samples, as well as the extraction solvents and volumes for sediment, and extraction salts and purification materials for shrimp. The optimized pretreatment conditions for different matrixes were as follows.

#### 2.4.1. Water

A mass of 0.25 g Na_2_EDTA was added to 250 mL of a water sample, with the pH being adjusted to 3. The target analytes in the samples were then concentrated using an HLB solid-phase extraction column, which had been activated with 5 mL of methanol and 5 mL of ultrapure water. Subsequently, the pretreated water sample was pumped through the HLB column at a flow rate of 5 to 8 mL/min. After sampling, the HLB column was rinsed with 6 mL of methanol, then dried with a vacuum pump, and the target analytes were eluted with 10 mL of a solution comprising 5 mmol/L ammonium acetate in methanol. The eluent was dried in a constant stream of nitrogen, then diluted to a volume of 1.0 mL with methanol. The solution was filtered through a 0.22 μm nylon filter membrane and transferred to an injection vial for subsequent analysis.

#### 2.4.2. Sediment

A freeze-dried sediment sample (2 g) with 20 ng internal standards was placed in a 50 mL plastic centrifuge tube. Subsequently, 10 mL of a 0.05 mol/L Na_2_EDTA aqueous solution at pH 3 was added to the tube and the solution was vortexed for 30 s. Next, 10 mL of a 5 mmol/L ammonium acetate–0.1% formic acid–acetonitrile solution was added and the mixture was shaken for 10 min. Then, 1 g of NaCl and 1 g of MgSO_4_ were added to the mixture and shaking was continued for 5 min. The turbid solution was subjected to centrifugation for 5 min at 4000 rpm. A volume of 5 mL of the transparent solution from the upper layer was then aspirated and concentrated to dryness under a steady flow of nitrogen, after which it was re-dissolved with the addition of 1 mL of methanol. The final volume was filtered through a 0.22 μm nylon membrane and transferred to the injection vial for further analysis.

#### 2.4.3. Shrimp

Prior to the pretreatment, the shrimp were defrosted at room temperature and the sample was homogenized using a grinder. A sample of shrimp (5 g) spiked with 20 ng internal standard was placed in a 50 mL centrifuge tube and was shaken for 10 min after the addition of 20 mL of 0.1% formic acid–5 mmol/L ammonium acetate–acetonitrile solution, 1 g NaCl and 1 g Na_2_SO_4_. Subsequently, 0.1 g of MWCNTs were added to the mixture, which was then shaken for a further five minutes. Next, the mixture was centrifuged at 4000 rpm for 5 min and 10 mL of supernatant was collected. The subsequent procedures were the same as those used for the pre-treatment of the sediment.

### 2.5. Instrumental Analysis

The chromatographic separation of the target compounds was conducted on a SHIMADZU Shim-pack GIST C_18_ column (2.1 × 100 mm, 2 μm) utilizing a Waters ACQUITY UHPLC system. The injection volume was 5 μL and the column temperature was 30 °C. The separation of PFASs and FQs was conducted with methanol (mobile phase A) and 5 mmol/L ammonium acetate–0.1% formic acid–water solution (mobile phase B) using a gradient elution program at a flow rate of 0.3 mL/min. The elution program was as follows: 0–1 min, 60% B; 1–15 min, 60%–10% B; 15–20 min, 10% B; 20–20.1 min, 10%–60% B; 20.1–25 min, 60% B.

An AB Sciex Triple-QuadTM 5500 mass spectrometer (Foster City, CA, USA) equipped with an electrospray ion source was used for qualitative and quantitative analysis of the target analytes. In electrospray ionization source positive ion mode (ESI+), the ion spray voltage (IS) was set at +4500 V. The declustering potential (DP), entrance potential (EP), and collision cell exit potential (CXP) were set at 55 V, 10 V, and 12 V, respectively. In the electrospray ionization source negative ion mode (ESI−). The IS was set at −4500 V, as well as the DP, EP, and CXP were set at −80 V, −10 V, and −13 V, respectively. The heater temperature (TEM) was set at 500 °C. The pressure of curtain gas (CUR), collision gas (CAD), spray gas (GS1), and auxiliary gas (GS2) were set at 35, 8, 50, and 50 (psi), respectively. The collision energies (CE), the qualitative ions, and other information on the target analytes are listed in Table 1 and Table S2, and the relevant data on the internal standards of 3 FQs and 8 PFASs are provided in Tables S3 and S4.

### 2.6. Method Validation

A series of matrix blank standard working solutions of PFAS and FQs in a range of concentrations (0.1, 0.5, 1.0, 5.0, 10.0, 50.0, and 100.0 μg/L) were prepared using purified water for the quantification of water samples. For sediment and shrimp samples, mixed standard solutions containing 30 target analytes were diluted with methanol into a series of mixed standard solutions at concentrations of 0.5, 1, 2, 5, and 10, and isotopic internal standard solutions of FQs and PFASs were added at concentration of 10 μg/L. Measurements were carried out under the optimal instrumental analytical conditions. The external standard curves and the internal standard curves for the target analytes were generated using MultiQuant^TM^ 3.0.2 software. The developed methods were validated for the linearity (*R*^2^), limit of detection (LOD), limit of quantification (LOQ), recovery, accuracy, precision, and matrix effect (ME).

## 3. Results and Discussion

### 3.1. Optimization of UHPLC-MS/MS Conditions

Standards were injected into the mass spectrometer using a syringe pump to optimize the precursor and product ions for 30 target analytes. Parameters such as declustering potential, entrance potential, and collision energy were optimized in the multi-reaction monitoring (MRM) mode to improve sensitivity. The optimal analytical parameters are described in Section 2.5.

PFASs are acidic compounds containing carboxylic or sulphonic acid groups in which the hydrogen atoms have been replaced by fluorine atoms. The detection of PFASs was performed in negative ionization mode because of the formation of [M-H]^−^ in the electrospray ion source. The precursor [M-H]^−^ ions were collapsed in the collision cell. The results showed that PFASs containing carboxylic acid groups tend to lose the neutral fragment CO_2_ to produce the fragment ion [M-H-44]^−^, while PFASs containing sulphonic acid groups can produce fragment ions [SO_3_]^−^. For a few compounds, the C-C bond or the C-S bond may be broken. For example, fragment ions [C_8_F_17_]^−^ can be observed in the mass spectra of NMeFOSAA and NEtFOSAA.

FQs are usually measured in positive ionization mode as they tend to acquire protons to form the precursor ion [M+H]^+^. The collision of the precursor ions produced product ions with loss of neutral fragments, H_2_O and CO_2_, consistent with the presence of the carboxylic acid group in the structure. If there was a piperazine ring substituent at the C-7 position of the core structure, rearranged characteristic peaks with loss of the neutral fragment C_2_H_5_N, C_3_H_7_N, or C_4_H_9_N would be observed after collision induced dissociation. The cleavage of the carbon ring formed at the C-1 and C-8 positions in the structure of flumequine results in the loss of the C_3_H_6_ fragment to give the product ion [M+H-H_2_O-C_3_H_6_]^+^. The information of the MS/MS fragments for the 30 target analytes has been listed in Table S2.

The conditions used for the liquid chromatographic separations were slightly adapted from those described in other publications [53,54]. As reported in those studies, the response intensity and peak shape were superior when methanol was used as the organic phase in comparison to acetonitrile. Accordingly, methanol was selected as the organic phase (mobile phase A) in subsequent experiments. The performances of separation were evaluated when the aqueous phase (mobile phase B) was comprised of (1) 0.1% formic acid aqueous solution, (2) 5 mmol/L ammonium acetate aqueous solution, and (3) aqueous solution with both 5 mmol/L ammonium acetate and 0.1% formic acid. Herein, a total of 30 typical fluorinated emerging pollutants were analyzed. When mobile phase B was set to 0.1% formic acid aqueous solution, the peak shapes of FQs were observed to be satisfactory, but the peaks of a few PFASs were noted to be of a poor quality. The use of a 5 mmol/L ammonium acetate aqueous solution resulted in a good response for PFASs, while the peak shapes of some FQs were found to be unsatisfactory. When the aqueous solution with both 5 mmol/L ammonium acetate and 0.1% formic acid was selected as the mobile phase B, the superior peak shapes and signal intensities of both PFASs and FQs were observed. Thus, 5 mmol/L ammonium acetate–0.1% formic acid aqueous solution was ultimately selected as the optimal aqueous phase in this study.

**Figure 1:**
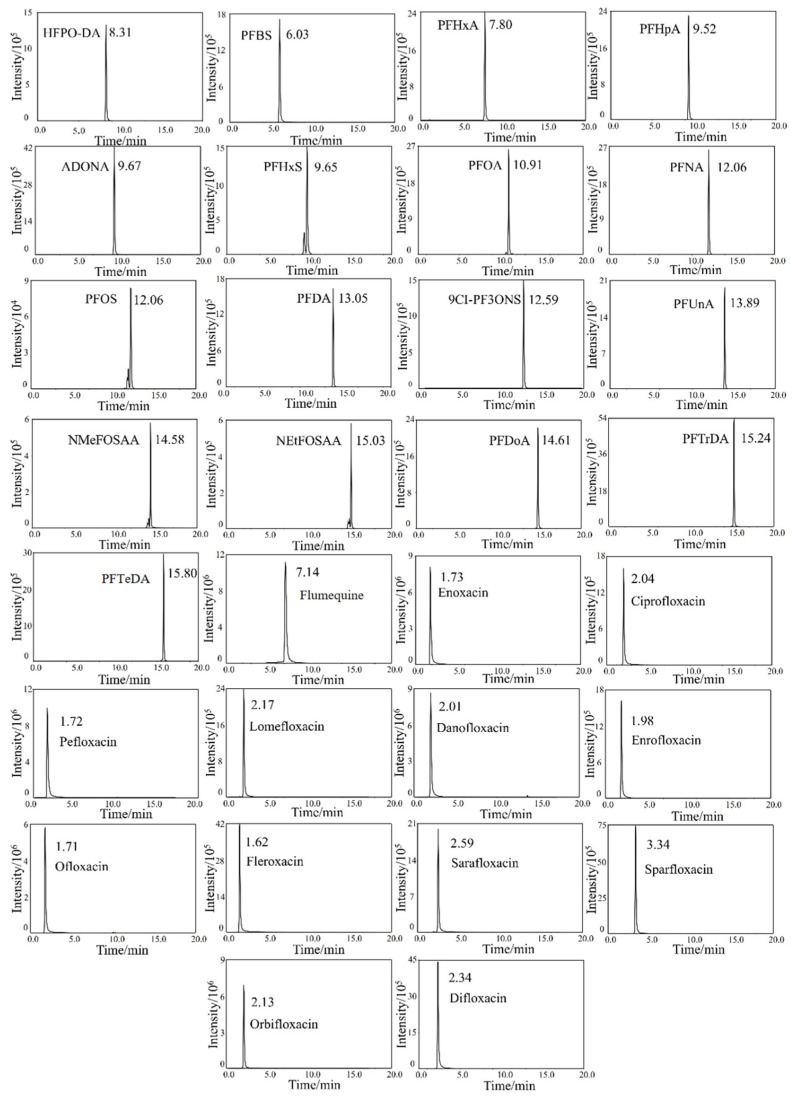
The extracted ion chromatograms of 17 PFASs and 13 FQs with a Shim-pack GIST C_18_ column.

The influence of different columns on the separation of target analytes was further examined, including (1) a Shim-pack GIST C_18_ column (100 × 2.1 mm, 2.0 μm, Shimadzu), (2) a CORTECS UHPLC C_18_ column (2.1 × 100 mm, 1.6 μm, Waters), and (3) a CORTECS UHPLC C_18_ column (2.1 × 100 mm, 1.7 μm, Waters). The experimental results showed that the peak profiles and response for the 30 target compounds were enhanced when performed on a Shim-pack GIST C_18_ column for separation. The extracted ion chromatograms obtained under optimal conditions are illustrated in Figure 1. It can be seen that the peak shapes for the 30 targeted fluorinated emerging pollutants are of high regularity in general, except for a few compounds (PFOS, PFHxS, NMeFOSAA, and NEtFOSAA) whose ion chromatograms possess small noise peak. The smaller peaks may be isomers of the compounds (branched compounds), which have been commonly observed in previous similar publications and were acceptable [54,55].

### 3.2. Optimization of Extraction and Purification Conditions

#### 3.2.1. Optimization of Solid Phase Extraction (SPE) Conditions for Water Samples

**Figure 2:**
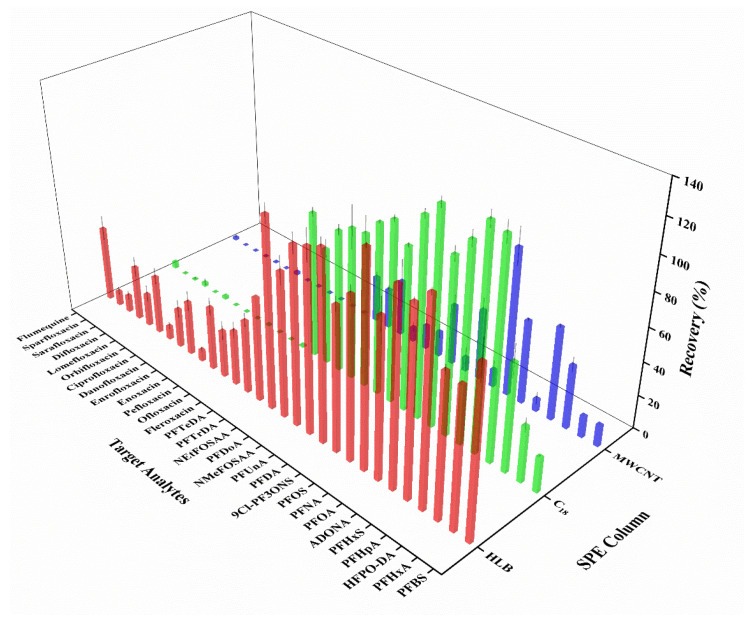
Average recoveries of 30 target analytes in water processed with different SPE columns: red columns for HLB, green columns for C_18_, and blue columns for MWCNT (Mean ± STD, *n* = 3).

Due to the relatively low levels in water, fluorinated emerging pollutants in water samples need to be concentrated by SPE columns. In this study, three types of SPE columns (HLB, C_18_, and MWCNT) were evaluated for investigating their capacity in enriching the target analytes. As shown in Figure 2, the recoveries of PFASs were superior on the HLB and C_18_ columns in comparison to those on the MWCNT column. Additionally, it was observed that FQs were poorly retained when using C_18_ or MWCNT columns. In contrast, all the target analytes were enriched and could be detected when using HLB columns. The C_18_ column with hydrophobic packing was used to separate non-polar compounds such as PFASs. Polar compounds such as FQs were difficult to retain on the C_18_ column, resulting in lower recoveries. The MWCNT column had strong selectivity for the adsorption of specific compounds and was weakly selective for FQs and PFASs. The HLB column was a universal SPE column packed with hydrophilic-ester friendly-balanced copolymers. It had good performance for sorption of both polar and non-polar compounds. Thus, the HLB column was selected for the pre-treatment of the water samples.

**Figure 3:**
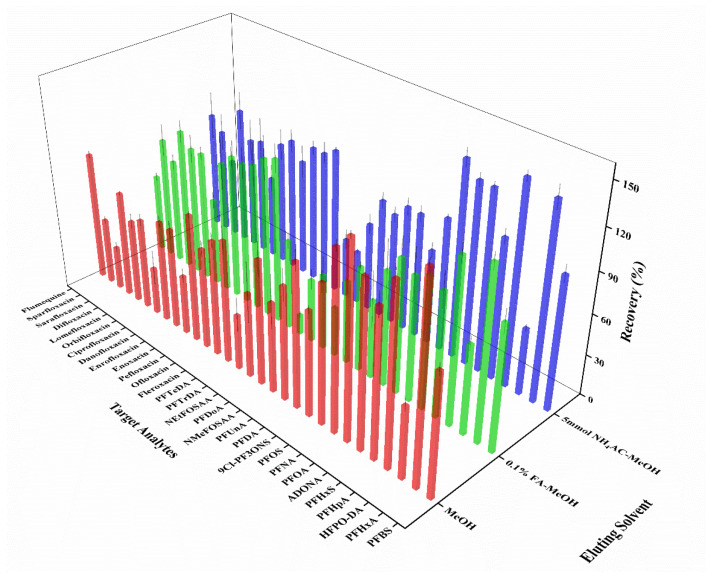
Average recoveries of 30 target analytes in water samples on SPE columns with different eluents: red columns for MeOH, green columns for 0.1% FA-MeOH, and blue columns for 5 mmol/L NH_4_AC-MeOH (Mean ± STD, *n* = 3).

The choice of eluting solvent has a significant impact on the recoveries of target compounds. Therefore, the effects of (1) methanol (MeOH), (2) 0.1% formic acid–methanol (0.1% FA-MeOH), and (3) 5 mmol/L ammonium acetate–methanol (5 mmol/L NH_4_AC-MeOH) as eluent on the extraction efficiency were investigated in subsequent experiments. The results showed that the use of MeOH as the eluent was ineffective for the elution of FQs since the recoveries of four FQs were below 50% (Figure 3). The addition of 0.1% FA led to an increase in the recoveries of FQs (59.3–122%), while this was accompanied by a decrease in the recoveries of PFASs, including 9Cl-PF3ONS (58.1%), PFDoA (13.9%), NEtFOSAA (8.0%), and NMeFOSAA (1.9%). Remarkably, as shown in Figure 3, the use of 5 mmol/L NH_4_AC-MeOH as the eluting solvent could improve the recoveries of FQs without affecting the elution efficiency of PFASs. This might be ascribed that the 5 mmol/L NH_4_AC-MeOH enabled the simultaneous dissociation of weak acid ions and weak base ions, resulting in the destruction of the strong ion-exchange interactions between the target analytes and the column fillers. Then the volume of eluting solvent was optimized by using 6 mL, 10 mL, and 14 mL of the 5 mmol/L NH_4_AC-MeOH solution, respectively. As shown in Figure S1, the target analytes retained on the HLB column were almost completely eluted at the volume of 10 mL. Therefore, 10 mL of 5 mmol/L NH_4_AC-MeOH was selected as the optimal eluent for subsequent experiments.

**Figure 4:**
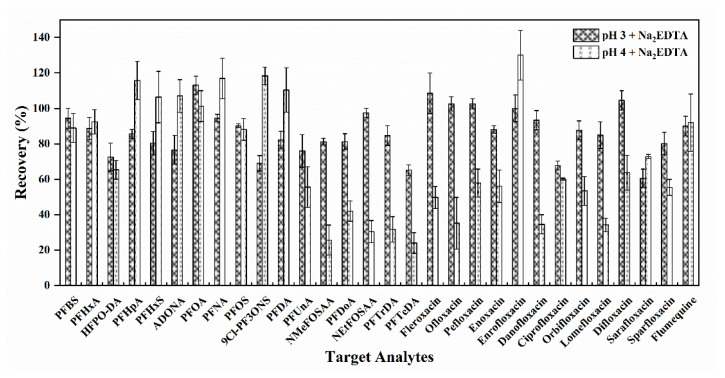
Average recoveries of 30 target analytes in water samples with the addition of Na_2_EDTA at different pH (Mean ± STD, *n* = 3).

Despite the improvement in the recoveries of the target analytes after the optimization of the SPE conditions, the recoveries of FQs still remained at a relatively low level (34.2–61.9%). The structures of FQs contain both acidic and basic functional groups. Consequently, the pH of the solution in which FQs are dissolved may result in a change of molecular state, with the potential to affect the recoveries. Moreover, since heavy metals in water can interact with antibiotics, Na_2_EDTA has been frequently used to complex with heavy metals in the pre-treatment, which can efficiently improve the recoveries [56,57]. Thus, the retention behavior of FQs in the extraction process was optimized by adjusting the state of the water samples. It has been demonstrated in previous research that FQs exist predominantly as amphoteric ions at pH 3–4, exhibiting structural stability and the highest intensity [56,58]. Accordingly, the experiments were conducted to evaluate the recoveries of the target fluorinated compounds in water samples with the addition of Na_2_EDTA at pH 3 and pH 4, respectively. As shown in Figure 4, the recoveries of all target analytes ranged from 60.0% to 130% at pH 3, while 14 compounds were observed with recoveries under 60% at pH 4. Thus, the water sample with the addition of Na_2_EDTA at pH 3 was chosen for optimal extraction.

#### 3.2.2. Optimization of Extraction Conditions for Sediment Samples

The extraction and clean-up of sediment and shrimp samples were performed using the QuEChERS method. QuEChERS technique has been proved to be an effective approach for the extraction of target analytes and the removal of interfering substances from complex matrices. The presence of heavy metals in the sediment may result in the formation of complexes with the target analytes, potentially impacting the efficiency of the extraction. Thus, based on the optimal conditions described in Section 3.2.1, 10 mL of Na_2_EDTA solution at pH 3 was added to 2 g of sediment for the purpose of releasing the target analytes and improving the efficiency of the pretreatment process.

**Figure 5:**
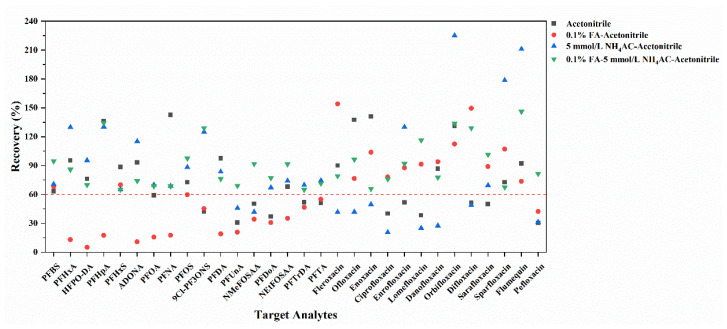
Average recoveries of 30 target analytes in sediment samples with different extraction solvent (Mean ± STD, *n* = 3). Red dashed line represents the recovery of 60%.

For the optimization of solvent for extraction, the recoveries of the targets were measured under the conditions of (1) acetonitrile, (2) 0.1% FA–acetonitrile, (3) 5 mmol/L NH_4_AC–acetonitrile, and (4) 0.1% FA-5 mmol/L NH_4_AC–acetonitrile as the extraction solvents, respectively. As can be seen in Figure 5, in comparison to the pure acetonitrile, 0.1% FA–acetonitrile was observed to be more effective for the extraction of FQs, with recoveries ranging from 43.0% to 154%. However, it showed poor performance for the extraction of PFASs. In contrast, the use of 5 mmol/L NH_4_AC–acetonitrile proved more effective for the extraction of PFASs, but the recoveries were relatively low for FQs. Notably, the acetonitrile solution containing both 0.1% FA and 5 mmol/L NH_4_AC provided superior performance in the extraction of all the 30 target analytes with the recoveries higher than 60%. Thus, 0.1% FA–5 mmol/L NH_4_AC–acetonitrile solution was chosen as the optimal extraction solvent for the sediments in the subsequent experiments.

Next, the impact of solvent volume on recoveries was assessed. The results in Figure S2 showed that the recoveries of all targets were above 60% for 2 g of sediment sample when the volume of extraction solvent was set at 10 mL. Upon increasing the volume to 20 mL, no significant difference in recoveries was observed for the majority of the analytes. However, for NMeFOSAA, PFTrDA, lomefloxacin, orbifloxacin, sparfloxacin, and pefloxacin, the abnormally high recoveries were observed with the increasing volume of the extraction. This can be attributed to the fact that the increase in solvent volume resulted in an enhancement of the matrix effects, which led to the change in recoveries. Thus, 10 mL was chosen as the optimal volume for the extraction of sediments.

#### 3.2.3. Optimization of Extraction and Purification Conditions for Shrimp Samples

In reference to the extraction method for sediments detailed in Section 3.2.2, with minor modifications, 5 g (wet weight, ww) of shrimp samples were processed using 20 mL of 0.1% FA–5 mmol/L NH_4_AC–acetonitrile as the extraction solvent. In the pretreatment of shrimp samples, the addition of salts is necessary for the extraction process due to high hydration and protein levels of the samples. Extraction salts can facilitate the transfer of the target analytes from the aqueous phase to the organic phase. The common dehydrating agents, MgSO_4_ and Na_2_SO_4_, can reduce the solubility of polar substances by salting out and enhance the extraction efficiency for the target compounds. The following experiment was conducted to compare the effects of extraction salts, 1 g NaCl + 1 g MgSO_4_ and 1 g NaCl + 1 g Na_2_SO_4_, on target analytes in shrimp samples. The addition of NaCl can assist in reducing the surface tension of the aqueous phase, thereby facilitating the separation of the organic and aqueous phases. The addition of MgSO_4_ or Na_2_SO_4_ enhanced the efficiency of the dehydration process and promoted the precipitation of proteins or other biomolecules that could potentially interfere with the analytes. As illustrated in Figure S3, the recoveries of PFASs were found to be lower when MgSO_4_ was used as the extraction salt in comparison to Na_2_SO_4_. This might be attributed to the influence of MgSO_4_ on the adsorption of PFASs and the higher solubility of Na_2_SO_4_ compared to MgSO_4_, which resulted in a more pronounced salting-out effect in the samples. The recoveries were similar for most FQs when a_2_SO_4_ or MgSO_4_ were used as the extraction salts. However, the recoveries of fleroxacin, ofloxacin, enrofloxacin, and pefloxacin were abnormally high (>180%) when MgSO_4_ was used, whereas the recoveries of the four targets ranged from 80% to 160% when using Na_2_SO_4_. This may be attributed to the fact that the matrix effects of fleroxacin, ofloxacin, enrofloxacin, and pefloxacin were enhanced in the MgSO_4_ system. In the Na_2_SO_4_ extraction salt system, strong matrix effects were also observed for some of the target analytes due to lack of purification, but the matrix effects were generally lower than that in the MgSO_4_ system. Therefore, 1 g NaCl + 1 g Na_2_SO_4_ was chosen as the extraction salt for shrimp samples.

**Figure 6:**
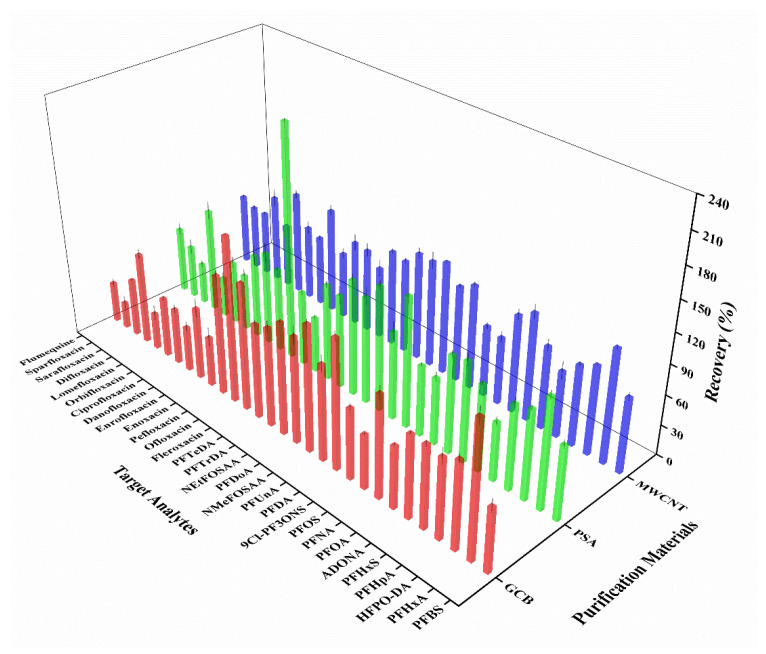
Average recoveries of 30 target analytes in shrimp samples pretreated with different purification materials: red columns for GCB, green columns for PSA, and blue columns for MWCNT (Mean ± STD, *n* = 3).

The recoveries of certain target analytes were observed to be anomalously high in the extraction as depicted in Figure S3. This phenomenon may be attributed to the presence of interfering substances, such as lipids and pigments, within the sample matrix. The effect of purification materials on the recoveries of analytes were therefore compared by adding 0.1 g of (1) graphitized carbon black (GCB), (2) ethylenediamine-N-propylsilane silica gel (PSA), and (3) carboxylated multi-walled carbon nanotubes (MWCNTs), respectively. The results showed that GCB had the best performance in removing pigments, followed by MWCNTs (Figure S4). However, as illustrated in Figure 6, the recoveries of the targets purified by MWCNTs were in the range of 65.2% to 113%, which were superior to those of GCB and PSA (Figure 6). Moreover, the samples purified by MWCNTs presented superior peak shapes during the separation, which was important for improving the resolution and quantitative accuracy of the analysis. Thus, MWCNTs were selected as the optimal purification materials for the pre-treatment of shrimp samples.

### 3.3. Method Validation

A series of quantitative parameters, including linearity, limit of detection (LOD), limit of quantification (LOQ), matrix effect (ME), recovery, accuracy, and precision, were evaluated under the optimal conditions to validate the developed methods. The results demonstrated that the ME ranged from 54.0% to 176.1% for water samples, 73.6% to 159.3% for sediment samples, and 44.9% to 154.2% for shrimp samples, respectively. It was also observed that some of the compounds had matrix effects, which may interfere with the detection. Thus, the target analytes were quantified by matrix-corrected standard curves for water samples and internal standard methods for sediment and shrimp samples.

**Figure 7:**
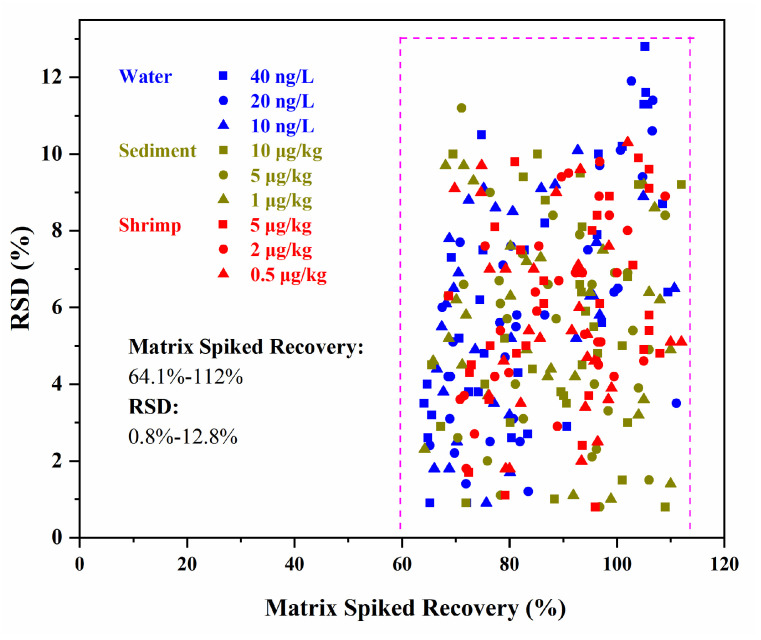
Results of matrix spiked recoveries and RSDs of the strategy (*n* = 6).

From the results as list in Tables S5–S7, *R*^2^ of the linear curves for all the target compounds were found to be greater than 0.99 within the range of 0.1–100 μg/L for water as well as 0.25–20 μg/L for sediment and shrimp. The LODs and LOQs of the target compounds in the water samples ranged from 0.01 to 0.30 ng/L and 0.04 to 1.00 ng/L, respectively. In the sediment, the LODs and LOQs of the target compounds ranged from 0.01 to 0.22 μg/kg and 0.03 to 0.73 μg/kg, respectively. The LODs of the target compounds for the shrimp samples were 0.01–0.23 μg/kg, with the LOQs ranging from 0.03 to 0.76 μg/kg. In the matrix spiked recovery experiments (Figure 7), the mean recoveries of the target analytes in water, sediment, and shrimp samples were found to be 64.1% to 111.1%, 65.5% to 112%, and 68.7% to 112%, respectively, with the relative standard deviations (RSDs) being lower than 12.8%. The results proved that the developed methods were satisfactory in terms of linearity, precision, and accuracy for the simultaneous detection of 30 fluorinated emerging pollutants in water, sediment, and shrimp samples.

### 3.4. Analysis of Fluorinated Emerging Pollutants in Various Samples from Shrimp Aquaculture Ponds

**Figure 8:**
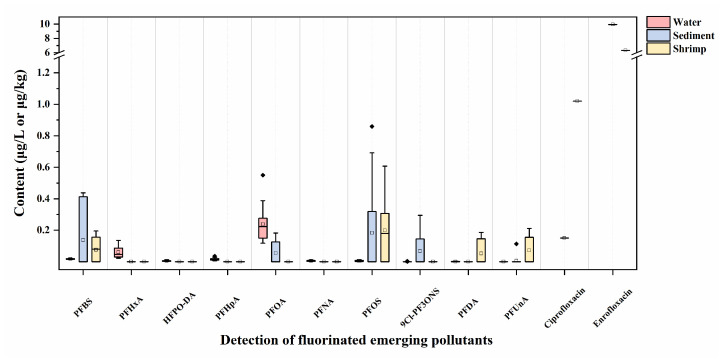
Contents of the fluorinated emerging pollutants in real samples from shrimp aquaculture ponds.

A total of 16 water, 13 sediment, and 19 shrimp samples from aquaculture ponds in Shanghai, eastern China, were analyzed to assess the applicability of the developed methods. The findings (Figure 8) indicated that PFASs constituted the primary fluorinated emerging pollutants in the shrimp aquaculture system since PFASs were detected in all the collected samples. In details, the concentrations of PFASs in the collected water samples ranged from 0.18 to 0.77 μg/L, with PFOA representing the dominant pollutant. Recent studies have reported the concentrations of PFASs in ambient surface waters in eastern China [59,60,61,62]. The presence of PFASs in rivers can be attributed to the direct or indirect discharges of human activities into the surrounding environment [61,63]. The water samples collected from the shrimp aquaculture ponds were derived from surface streams. The introduction of water may result in the migration of PFASs into the ponds. The relatively poor mobility of water in aquaculture ponds can lead to the accumulation of contaminants in the environment.

Sediment plays an important role in the migration and transformation of organic contaminants in the pond system. It represents a critical component of the aqueous environment, providing a hydrophobic medium for pollutants. Additionally, sediments, which are habitats for benthic organisms, can serve as sites for the deposition and storage of PFASs as reported before [64,65]. The concentrations of PFASs in the sediment ranged from 0.13 to 1.41 μg/kg (dry weight, dw), with PFBS, PFOA, PFOS, and 9Cl-PF3ONS as the main species. In contrast to the water samples, the dominant PFASs in the sediment samples were PFOS and PFBS. PFUnA was identified in one sample at a concentration of 0.11 μg/kg (dw). As anionic surfactants, PFASs featured a combination of polar hydrophilic groups (such as carboxyl groups and sulfonic acid groups) and non-polar hydrophobic groups (carbon chains), which enabled their solubility and detachment from aqueous phases [66]. A study of the adsorption of PFOS from sediment to water revealed that sediment showed superior adsorption capacity for PFOS compared to water [67]. Moreover, PFASs with a carbon chain length exceeding 11 were exclusively identified in sediments, not in the water in Tokyo Bay, Japan [67]. The findings indicated that both carbon chain length and functional groups can exert an influence on the concentration of PFASs in the sediment.

The characteristics of thermal stability, chemical stability, and difficulty in degradation of PFASs resulted in these compounds being easily accumulated and frequently detected in aquatic organisms [68,69]. The transfer of PFASs from water and sediment to shrimps can occur via the food chain. In this work, the concentrations of PFASs in the shrimp samples were found to be in the range of 0.09–0.96 μg/kg. The contaminants detected were PFBS, PFOS, PFNA, and PFUnA, with notable variations in the proportions of each contaminant observed among shrimp samples collected from distinct sampling sites. The findings showed that the total concentration of PFBS and PFOS was generally higher than that of PFUnA and PFDA in the majority of shrimp samples. This observation was consistent with those reported in other studies [70,71]. In addition, PFOS was detected in more than half of the samples, suggesting the possibility of historical residual contamination in the sediment. This indicated that residual PFOS can still affect organisms and human health via the environment, despite the current prohibition of its use.

The application of FQs has greatly diminished as a consequence of the stringent regulation of the utilization of these antibiotics in the aquaculture in Shanghai over recent years. Thus, it is not common for FQs to be contaminated in the shrimp aquaculture system. Herein, the concentration of FQs in all water samples was found to be below the LODs collected from the shrimp ponds. Only in one same sampling pond, two types of FQs, ciprofloxacin and enrofloxacin, were found in the sediment and shrimp samples with concentrations of 0.15 µg/kg and 9.94 µg/kg in the sediment, and 1.02 µg/kg and 6.31 µg/kg in the shrimp, respectively. This may be attributed to the uptake and bioaccumulation of FQs in the shrimps from aquatic systems, which may deserve further attention in future.

## 4. Conclusions

In this work, sensitive and high-throughput methods for the simultaneous analysis of 30 typical fluorinated emerging pollutants were developed. By combining SPE and QuEChERS with UHPLC-MS/MS, 17 PFASs and 13 FQs in different matrixes from shrimp aquaculture system were simultaneously detected and quantitated. The developed methods obviated the requirement for two pre-treatment stages compared to the traditional methods, which resulted in considerable advantages in solvent and time consumption savings. Finally, the high-throughput analytical strategy was successfully applied in the simultaneous analysis of 16 water, 13 sediment, and 19 shrimp samples. PFASs were detected in all samples, with concentration ranges of 0.18–0.77 μg/L in water, 0.13–1.41 μg/kg (dry weight) in sediment, and 0.09–0.96 μg/kg (wet weight) in shrimp, respectively. Ciprofloxacin and enrofloxacin were found in the one sediment sample and one shrimp sample. The findings indicated that contamination with PFASs was present in all samples to varying degrees, whereas contamination with FQs was only identified in the sediment and shrimp samples of one sampled pond.

## Figures and Tables

**Table 1:** The parameters of UHPLC-MS/MS for the 30 target analytes.

Compound	Retention Time (min)	Precursor Ion	Product Ion	Collision Energy/V
PFBS	6.03	299.0	80.0	−33
PFHxA	7.80	313.0	269.0	−11
HFPO-DA	8.31	329.0	285.0	−8
PFHpA	9.52	363.0	319.0	−10
PFHxS	9.65	399.0	80.0	−32
ADONA	9.67	377.0	251.0	−12
PFOA	10.91	413.0	369.0	−10
PFNA	12.06	463.0	419.0	−12
PFOS	12.06	499.0	80.0	−42
9Cl-PF3ONS	12.59	531.0	351.0	−24
PFDA	13.05	513.0	469.0	−10
PFUnA	13.89	563.0	519.0	−10
NMeFOSAA	14.58	570.0	419.0	−20
PFDoA	14.61	613.0	569.0	−10
NEtFOSAA	15.03	584.0	419.0	−20
PFTrDA	15.24	663.0	619.0	−14
PFTeDA	15.80	713.0	669.0	−12
Fleroxacin	1.62	370.4	326.4 */269.4	30/40
Ofloxacin	1.71	362.4	318.4 */261.3	30/40
Pefloxacin	1.72	334.1	316.1 */290.2	27/25
Enoxacin	1.73	321.1	303.4 */232.2	35/48
Enrofloxacin	1.98	360.6	316.4 */245.4	30/40
Danofloxacin	2.01	358.3	340.3 */283.4	30/40
Ciprofloxacin	2.04	332.4	288.3 */245.3	25/33
Orbifloxacin	2.13	396.3	352.3 */295.4	27/35
Lomefloxacin	2.17	352.3	308.4 */265.4	28/33
Difloxacin	2.34	400.4	356.2 */299.3	28/42
Sarafloxacin	2.59	386.4	342.3 */299.2	28/43
Sparfloxacin	3.34	393.3	349.4 */292.4	30/38
Flumequine	7.14	262.3	244.3 */202.3	30/49

*: quantitative ion

## Data Availability

The original contributions presented in the study are included in the article/Supplementary Materials, further inquiries can be directed to the corresponding author.

## Supplementary Materials

The following supporting information can be downloaded at: https://www.mdpi.com/article/10.3390/foods13203286/s1, Figure S1: Average recoveries of the extraction of 30 target analytes in water samples with different volumes; Figure S2: Average recoveries of the extraction of 30 target analytes in sediment samples with different solvent volumes; Figure S3: Average recoveries of 30 target analytes in biological samples pretreated with different extraction salts; Figure S4: Performance of different purification materials in removing pigments from biological samples; Table S1: Information on the collected samples; Table S2: Information on the formula, structure, and MS/MS fragments of 30 target fluorinated emerging pollutants; Table S3: Information on the eight PFASs’ internal standards and their quantitative compounds for sediment and shrimp samples; Table S4: Information on the three FQs’ internal standards and their quantitative compounds for sediment and shrimp samples; Table S5: Summary of linearity, LODs, LOQs, and ME of 30 target analytes in water samples; Table S6: Summary of linearity, LODs, LOQs, and ME of 30 target analytes in sediment samples; Table S7: Summary of linearity, LODs, LOQs, and ME of 30 target analytes in shrimp samples.
