# Supplementary material for: Simultaneous and High-Throughput Analytical Strategy of 30 Fluorinated Emerging Pollutants Using UHPLC-MS/MS in the Shrimp Aquaculture System
Source: Foods. 2024 Oct 16;13(20):3286. doi: 10.3390/foods13203286 (PMC11507328; doi:10.3390/foods13203286)
Supplement: Supplementary file 1 [file foods-13-03286-s001.zip › Supplementary Material.pdf]

Supplementary Material

Simultaneous and high-throughput analytical strategy of 30 fluorinated emerging pollutants using UHPLC-MS/MS in the shrimp aquaculture system

Di Huang [1,2], Chengbin Liu [1,2,3], Huatian Zhou [1,4], Xianli Wang [1,2], Qicai Zhang [1,2], Xiaoyu Liu [1], Zhongsheng Deng [1], Danhe Wang [1,2], Yameng Li [1,2], Chunxia Yao [1,2], Weiguo Song [1,2,3,4,*], Qinxiong Rao [1,2]

[1] The Institute of Agro-food Standards and Testing Technology, Shanghai Academy of Agricultural Sciences, Shanghai, 201403, China

[2] Key Laboratory of Food Quality Safety and Nutrition (Co-construction by Ministry and Province), Ministry of Agriculture and Rural Affairs, Shanghai 201403, China

[3] Shanghai Co-Elite Agri-food Testing Technical Service Co., Ltd, Shanghai 201403, China

[4] School of Health Science and Engineering, University of Shanghai for Science & Technology, Shanghai, 100049, China

* Correspondence: songweiguo@saas.sh.cn (W.S.); Tel.: +86-21622-02796

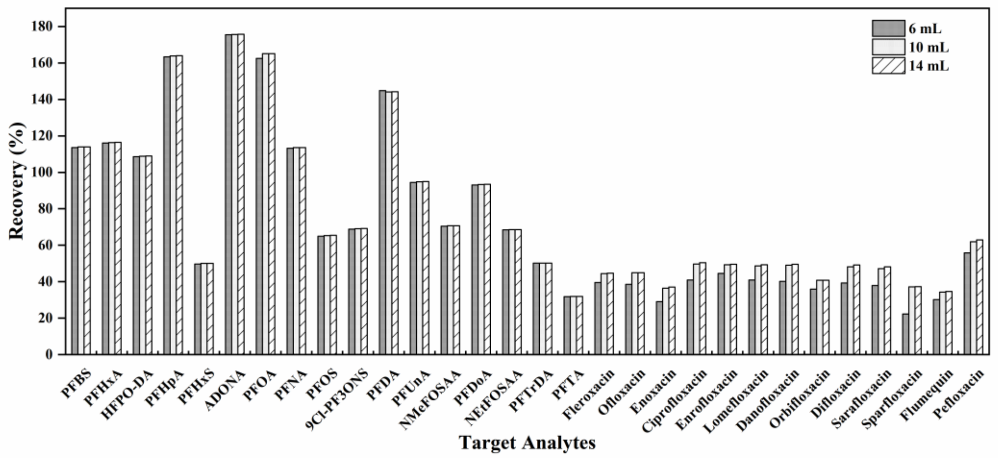

Figure S1. Average recoveries of the extraction of 30 target analytes in water samples with different volumes

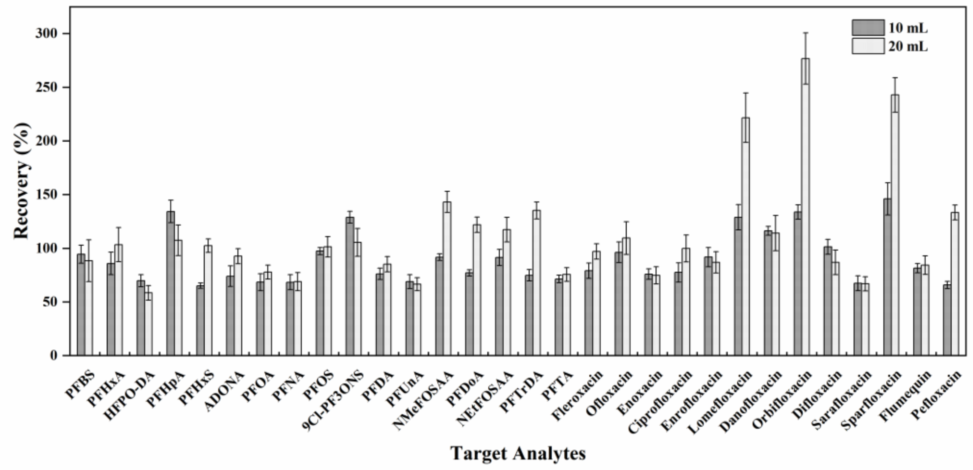

Figure S2. Average recoveries of the extraction of 30 target analytes in sediment samples with different solvent volumes

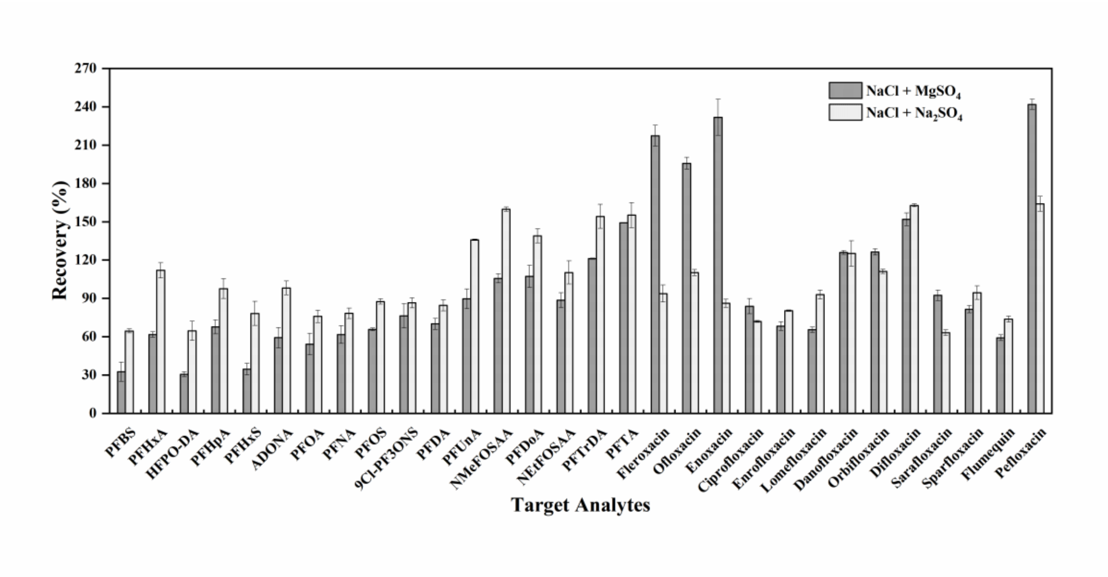

Figure S3. Average recoveries of 30 target analytes in biological samples pretreated with different extraction salts

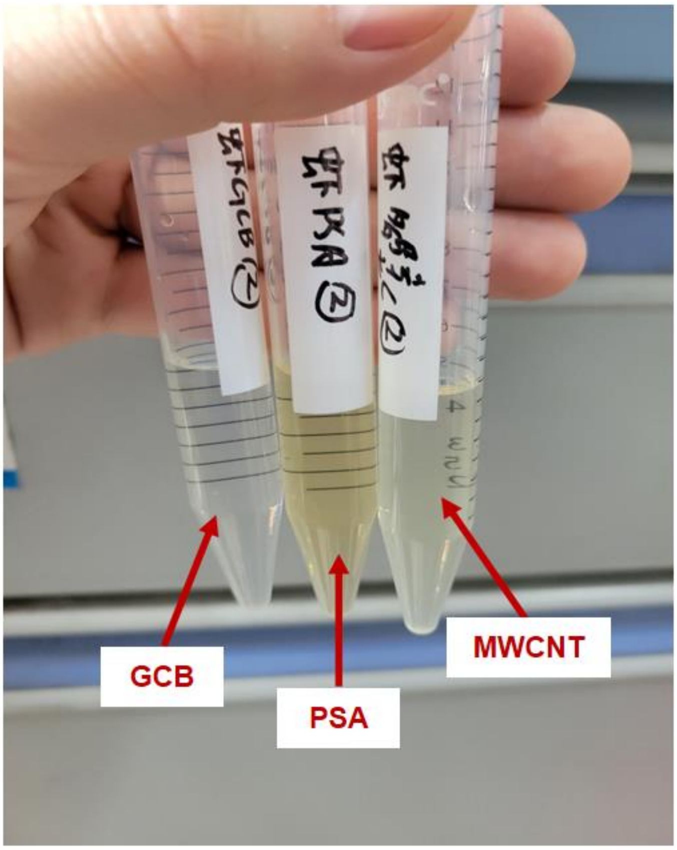

Figure S4. Performance of different purification materials in removing pigments from biological samples

Table S1. Information on the collected samples

Ponds No.	Date	Coordinates	Sample types
1	2023.08.03	121.408261 E，30.819838 N	Water、Sediment、Shrimp
2	2023.08.03	121.413775 E，30.812185 N	Water、Sediment、Shrimp
3	2023.08.03	121.418621 E，30.815406 N	Water、Sediment、Shrimp
4	2023.08.03	121.397416 E，30.819549 N	Water、Sediment、Shrimp
5	2023.08.03	121.380104 E，30.825272 N	Water、Sediment、Shrimp
6	2023.08.08	121.384848 E，30.867297 N	Sediment、Shrimp
7	2023.08.16	121.388234 E，30.865855 N	Water、Sediment、Shrimp
8	2023.08.29	121.837304 E，31.582802 N	Water、Shrimp
9	2023.09.01	121.649727 E，31.548989 N	Water、Sediment、Shrimp
10	2023.09.01	121.664962 E，31.647293 N	Water、Shrimp
11	2023.09.01	121.667037 E，31.646427 N	Water、Sediment、Shrimp
12	2023.09.08	121.267370 E，30.872697 N	Water、Sediment、Shrimp
13	2023.09.08	121.284219 E，30.860251 N	Water、Sediment、Shrimp
14	2023.09.15	121.961354 E，30.943509 N	Water、Sediment、Shrimp
15	2023.09.15	121.776601 E，31.022304 N	Shrimp
16	2023.09.15	121.910849 E，30.937269 N	Shrimp
17	2023.09.17	120.893757 E，31.096315 N	Water、Shrimp
18	2023.09.17	120.896766 E，31.093726 N	Water、Shrimp
19	2023.09.17	120.902872 E，31.093541 N	Water、Sediment、Shrimp

Table S2. Information on the formula, structure, and MS/MS fragments of 30 target fluorinated emerging pollutants

Compound	Formula	Structure	MS/MS fragments
PFBS	$C_4HF_9O_3S$	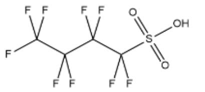	$[M-H]^- \rightarrow [SO_3]^-$<br>299.0> 80.0
PFHxA	$C_6HF_{11}O_2$	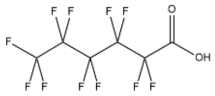	$[M-H]^- \rightarrow [M-H-CO_2]^-$<br>313.0> 269.0
HFPO-DA	$C_6HF_{11}O_3$	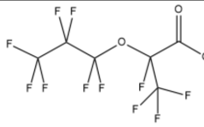	$[M-H]^- \rightarrow [M-H-CO_2]^-$<br>329.0> 285.0
PFHpA	$C_7HF_{13}O_2$	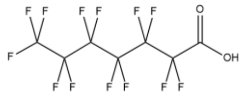	$[M-H]^- \rightarrow [M-H-CO_2]^-$<br>363.0> 319.0
PFHxS	$C_6HF_{13}O_3S$	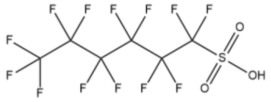	$[M-H]^- \rightarrow [SO_3]^-$<br>399.0> 80.0
ADONA	$C_7H_2F_{12}O_4$	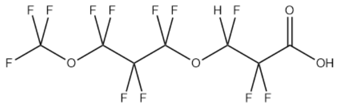	$[M-H]^- \rightarrow [C_4F_9O_2]^-$<br>377.0> 251.0
PFOA	$C_8HF_{15}O_2$	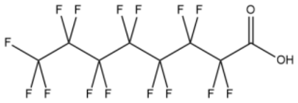	$[M-H]^- \rightarrow [M-H-CO_2]^-$<br>413.0> 369.0
PFNA	$C_9HF_{17}O_2$	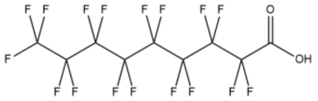	$[M-H]^- \rightarrow [M-H-CO_2]^-$<br>463.0> 419.0
PFOS	$C_8HF_{17}O_3S$	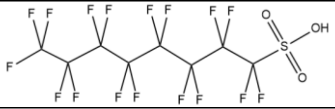	$[M-H]^- \rightarrow [SO_3]^-$<br>499.0> 80.0
9Cl-PF3ONS	$C_8HF_{16}ClO_4S$	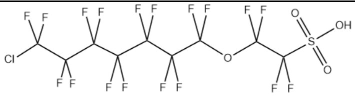	$[M-H]^- \rightarrow [C_6F_{12}ClO]^-$<br>531.0> 351.0
PFDA	$C_{10}HF_{19}O_2$	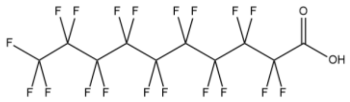	$[M-H]^- \rightarrow [M-H-CO_2]^-$<br>513.0> 469.0
PFUnA	$C_{11}HF_{21}O_2$	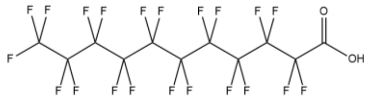	$[M-H]^- \rightarrow [M-H-CO_2]^-$<br>563.0> 519.0
NMeFOSAA	$C_{11}H_6F_{17}NO_4S$	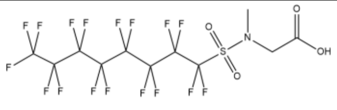	$[M-H]^- \rightarrow [C_8F_{17}]^-$<br>570.0> 419.0
PFDoA	$C_{12}HF_{23}O_2$	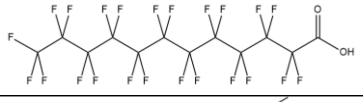	$[M-H]^- \rightarrow [M-H-CO_2]^-$<br>613.0> 569.0
NEtFOSAA	$C_{12}H_8F_{17}NO_4S$	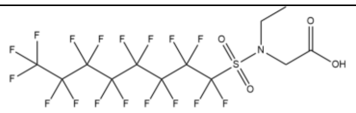	$[M-H]^- \rightarrow [C_8F_{17}]^-$<br>584.0> 419.0

PFTrDA	$C_{13}HF_{25}O_2$	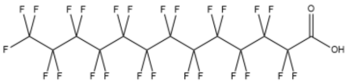	$[M-H]^- \rightarrow [M-H-CO_2]^-$<br>663.0> 619.0
PFTeDA	$C_{14}HF_{27}O_2$	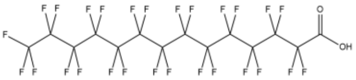	$[M-H]^- \rightarrow [M-H-CO_2]^-$<br>713.0> 669.0
Fleroxacin	$C_{17}H_{18}F_3N_3O_3$	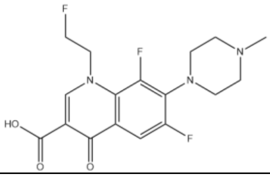	$[M+H]^+ \rightarrow [M+H-CO_2]^+$<br>370.4> 326.4*<br>$[M+H]^+ \rightarrow [M+H-CO_2-C_3H_7N]^+$<br>370.4> 269.4
Ofloxacin	$C_{18}H_{20}FN_3O_4$	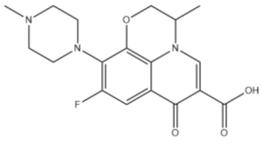	$[M+H]^+ \rightarrow [M+H-CO_2]^+$<br>362.4> 318.4*<br>$[M+H]^+ \rightarrow [M+H-CO_2-C_3H_7N]^+$<br>362.4> 261.3
Pefloxacin	$C_{17}H_{20}FN_3O_3$	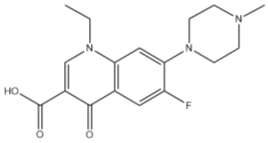	$[M+H]^+ \rightarrow [M+H-H_2O]^+$<br>334.1> 316.1*<br>$[M+H]^+ \rightarrow [M+H-CO_2]^+$<br>334.1> 290.2
Enoxacin	$C_{15}H_{17}FN_4O_3$	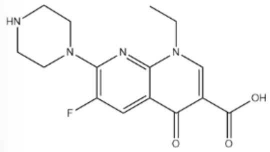	$[M+H]^+ \rightarrow [M+H-H_2O]^+$<br>321.1> 303.4*<br>$[M+H]^+ \rightarrow [M+H-H_2O-C_4H_9N]^+$<br>321.1> 232.2
Enrofloxacin	$C_{19}H_{22}FN_3O_3$	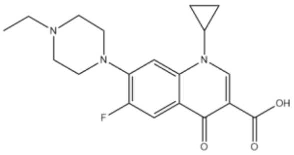	$[M+H]^+ \rightarrow [M+H-CO_2]^+$<br>360.6> 316.4*<br>$[M+H]^+ \rightarrow [M+H-CO_2-C_4H_9N]^+$<br>360.6> 245.4
Danofloxacin	$C_{19}H_{20}FN_3O_3$	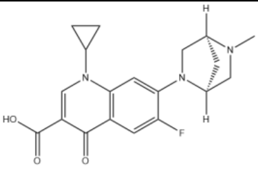	$[M+H]^+ \rightarrow [M+H-H_2O]^+$<br>358.3> 340.3*<br>$[M+H]^+ \rightarrow [M+H-H_2O-C_3H_7N]^+$<br>358.3> 283.4
Ciprofloxacin	$C_{17}H_{18}FN_3O_3$	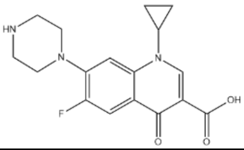	$[M+H]^+ \rightarrow [M+H-CO_2]^+$<br>332.4> 288.3*<br>$[M+H]^+ \rightarrow [M+H-CO_2-C_2H_5N]^+$<br>332.4> 245.3
Orbifloxacin	$C_{19}H_{20}F_3N_3O_3$	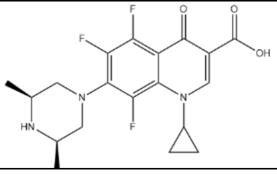	$[M+H]^+ \rightarrow [M+H-CO_2]^+$<br>396.3> 352.3*<br>$[M+H]^+ \rightarrow [M+H-CO_2-C_3H_7N]^+$<br>396.3> 295.4
Lomefloxacin	$C_{17}H_{19}F_2N_3O_3$	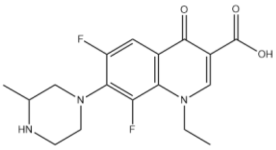	$[M+H]^+ \rightarrow [M+H-CO_2]^+$<br>352.3> 308.4*<br>$[M+H]^+ \rightarrow [M+H-CO_2-C_2H_5N]^+$<br>352.3> 265.4

Difloxacin	$C_{21}H_{19}F_2N_3O_3$	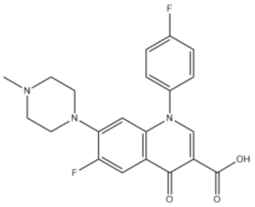	$[M+H]^+ \rightarrow [M+H-CO_2]$+<br>400.4> 356.2*<br>$[M+H]^+ \rightarrow [M+H-CO_2-C_3H_7N]^+$<br>400.4> 299.3
Sarafloxacin	$C_{20}H_{17}F_2N_3O_3$	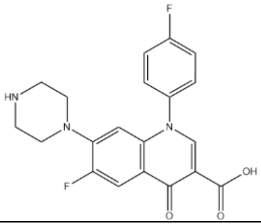	$[M+H]^+ \rightarrow [M+H-CO_2]^+$<br>386.4> 342.3*<br>$[M+H]^+ \rightarrow [M+H-CO_2-C_2H_5N]^+$<br>386.4> 299.2
Sparfloxacin	$C_{19}H_{22}F_2N_4O_3$	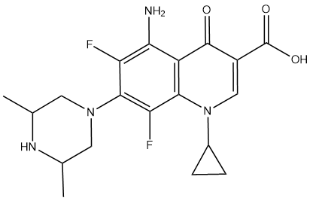	$[M+H]^+ \rightarrow [M+H-CO_2]$+<br>393.3> 349.4*<br>$[M+H]^+ \rightarrow [M+H-CO_2-C_3H_7N]^+$<br>393.3> 292.4
Flumequine	$C_{14}H_{12}FNO_3$	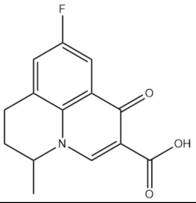	$[M+H]^+ \rightarrow [M+H-H_2O]^+$<br>262.3> 244.3*<br>$[M+H]^+ \rightarrow [M+H-H_2O-C_3H_6]^+$<br>262.3> 202.3

*：quantitative ion

Table S3. Information on the eight PFASs' internal standards and their quantitative compounds for sediment and shrimp samples

Internal Standard	Qualitative ion pairs (*m/z*)	Quantitative compounds
$^{13}C_4$ -PFOA	417.0, 372.0	PFOA
$^{13}C_5$ -PFNA	467.9, 422.9	PFNA
$^{13}C_2$ -PFDA	514.9, 469.9	PFDA
$^{13}C_2$ -PFUnDA	564.9, 519.9	PFUnA
$^{13}C_2$ -PFDoDA	614.9, 570.0	PFDoA, PFTrDA, PFTeDA
$^{18}O_2$ -PFHxS	402.9, 84.0	PFHxS
$^{13}C_4$ -PFOS	503.0, 80.0	PFBS, PFOS, 9Cl-PF3ONS, NMeFOSAA, NEtFOSAA

Table S4. Information on the three FQs' internal standards and their quantitative compounds for sediment and shrimp samples

Internal Standard	Qualitative ion pairs(*m/z*)	Quantitative compounds
Norfloxacin-D_5	325.3, 281.4*<br>325.3, 307.4	Fleroxacin, Ofloxacin, Pefloxacin, Enoxacin
Ciprofloxacin-D_8	340.5, 296.5*<br>340.5, 322.5	Lomefloxacin, Orbifloxacin, Danofloxacin, Difloxacin, Ciprofloxacin
Enrofloxacin-D_5	365.6, 321.4	Enrofloxacin, Sarafloxacin, Sparfloxacin, Flumequine

Table S5. Summary of linearity, LODs, LOQs, and ME of 30 target analytes in water samples

Compound	Regression equation (External)	R^2	LOD (ng/L)	LOQ (ng/L)	ME (%)
PFBS	$y = 2.816\times10^5x + 1.619\times10^5$	0.9994	0.02	0.05	145.2 ± 9.3
PFHxA	$y = 1.675\times10^5x - 5.504\times10^4$	0.9997	0.08	0.25	175.2 ± 8.6
HFPO-DA	$y = 7.922\times10^4x + 5.444\times10^3$	0.9997	0.09	0.29	171.9 ± 5.0
PFHpA	$y = 1.417\times10^5x - 2.302\times10^4$	0.9997	0.04	0.15	148.4 ± 6.9
PFHxS	$y = 1.974\times10^5x + 1.497\times10^4$	0.9991	0.17	0.57	129.0 ± 2.8
ADONA	$y = 2.798\times10^5x - 2.774\times10^4$	0.9997	0.02	0.07	157.3 ± 9.8
PFOA	$y = 1.572\times10^5x + 9.740\times10^3$	0.9997	0.06	0.22	176.1 ± 4.7
PFOS	$y = 9.888\times10^3x + 1.064\times10^4$	0.9976	0.30	1.00	149.3 ± 9.6
PFNA	$y = 1.653\times10^5x - 3.867\times10^3$	0.9997	0.03	0.10	153.2 ± 7.5
9Cl-PF3ONS	$y = 1.575\times10^5x + 1.129\times10^5$	0.9991	0.02	0.06	158.3 ± 4.4
PFDA	$y = 9.689\times10^4x - 3.298\times10^4$	0.9997	0.03	0.09	173.4 ± 2.2
NMeFOSAA	$y = 3.081\times10^4x - 7.366\times10^3$	0.9997	0.05	0.17	120.0 ± 8.1
PFUnA	$y = 8.579\times10^4x + 6.179\times10^3$	0.9997	0.02	0.06	157.5 ± 6.4
NEtFOSAA	$y = 2.192\times10^4x + 9.875\times10^3$	0.9997	0.05	0.18	111.9 ± 1.9
PFDoA	$y = 6.553\times10^4x + 2.780\times10^4$	0.9997	0.03	0.10	161.6 ± 4.9
PFTrDA	$y = 1.062\times10^5x + 1.509\times10^5$	0.9976	0.02	0.05	130.0 ± 2.4
PFTeDA	$y = 3.229\times10^4x + 9.333\times10^4$	0.9908	0.05	0.15	77.3 ± 3.3
Fleroxacin	$y = 2.618\times10^5x - 5.204\times10^3$	0.9990	0.04	0.12	71.7 ± 5.9
Ofloxacin	$y = 4.136\times10^5x + 4.901\times10^3$	0.9940	0.02	0.07	73.4 ± 1.9
Pefloxacin	$y = 9.353\times10^5x + 4.772\times10^4$	0.9922	0.03	0.10	89.9 ± 7.6
Enoxacin	$y = 7.097\times10^5x - 3.681\times10^4$	0.9986	0.04	0.12	77.9 ± 4.1
Enrofloxacin	$y = 1.185\times10^5x + 1.795\times10^3$	0.9953	0.07	0.23	72.8 ± 9.5
Danofloxacin	$y = 7.201\times10^5x - 2.793\times10^4$	0.9942	0.03	0.10	80.7 ± 5.3
Ciprofloxacin	$y = 9.362\times10^4x + 6.917\times10^3$	0.9998	0.07	0.25	81.0 ± 3.9
Orbifloxacin	$y = 4.017\times10^5x + 4.013\times10^4$	0.9962	0.01	0.04	69.7 ± 6.8
Lomefloxacin	$y = 1.294\times10^5x + 6.047\times10^3$	0.9914	0.02	0.07	74.1 ± 5.5
Difloxacin	$y = 2.927\times10^5x - 1.351\times10^4$	0.9973	0.03	0.10	70.5 ± 5.3
Sarafloxacin	$y = 1.051\times10^5x - 4.447\times10^3$	0.9947	0.04	0.12	71.5 ± 5.2
Sparfloxacin	$y = 4.625\times10^5x - 2.403\times10^3$	0.9959	0.01	0.04	67.2 ± 5.2
Flumequine	$y = 1.392\times10^6x + 2.109\times10^5$	0.9911	0.01	0.05	54.0 ± 7.4

Table S6. Summary of linearity, LODs, LOQs, and ME of 30 target analytes in sediment samples

Compound	Regression equation (Internal)	R^2	LOD (μg/kg)	LOQ (μg/kg)	ME (%)
PFBS	$y = 0.59154x + 0.01513$	0.9995	0.01	0.03	102.4 ± 1.2
PFHxA	$y = 0.07395x + 0.00418$	0.9984	0.02	0.07	121.3 ± 7.7
HFPO-DA	$y = 0.04789x + 0.00327$	0.9991	0.02	0.06	99.7 ± 5.7
PFHpA	$y = 0.04623x + 0.00318$	0.9991	0.01	0.03	140.5 ± 1.1
PFHxS	$y = 0.31981x + 0.01095$	0.9994	0.03	0.10	117.2 ± 8.6
ADONA	$y = 0.28190x + 0.00354$	0.9998	0.02	0.06	100.9 ± 6.1
PFOA	$y = 0.31285x + 0.00973$	0.9976	0.04	0.13	159.3 ± 6.7
PFOS	$y = 0.58471x + 0.01577$	0.9995	0.03	0.10	106.6 ± 7.9
PFNA	$y = 0.05022x + 0.00410$	0.9989	0.03	0.10	120.9 ± 9.1
9Cl-PF3ONS	$y = 0.01616x + 0.04192$	0.9993	0.05	0.18	98.2 ± 3.9
PFDA	$y = 0.11397x + 0.01488$	0.9978	0.02	0.06	107.6 ± 3.0
NMeFOSAA	$y = 0.05512x + 0.00892$	0.9990	0.02	0.06	104.5 ± 2.6
PFUnA	$y = 0.08975x + 0.05217$	0.9922	0.05	0.15	102.5 ± 8.8
NEtFOSAA	$y = 0.03411x + 0.00839$	0.9929	0.18	0.60	105.7 ± 2.6
PFDoA	$y = 0.05940x + 0.03850$	0.9948	0.12	0.37	101.6 ± 0.8
PFTrDA	$y = 0.02764x - 0.00281$	0.9985	0.10	0.33	99.7 ± 1.4
PFTeDA	$y = 0.01869x + 0.00496$	0.9997	0.15	0.44	107.3 ± 9.3
Fleroxacin	$y = 0.02823x + 0.01461$	0.9980	0.17	0.57	81.6 ± 2.9
Ofloxacin	$y = 0.03315x + 0.02804$	0.9975	0.20	0.67	85.4 ± 6.8
Pefloxacin	$y = 0.06899x - 0.01585$	0.9968	0.16	0.53	87.9 ± 1.2
Enoxacin	$y = 0.05320x - 0.03552$	0.9925	0.22	0.73	103.3 ± 5.4
Enrofloxacin	$y = 0.01273x + 0.00220$	0.9975	0.20	0.67	93.1 ± 9.2
Danofloxacin	$y = 0.17646x - 0.06410$	0.9999	0.18	0.59	89.9 ± 2.3
Ciprofloxacin	$y = 0.03607x + 0.00747$	0.9966	0.21	0.69	112.9 ± 7.2
Orbifloxacin	$y = 0.15420x - 0.06691$	0.9910	0.09	0.30	81.2 ± 0.5
Lomefloxacin	$y = 0.05620x + 0.00955$	0.9996	0.17	0.56	94.1 ± 3.2
Difloxacin	$y = 0.11201x + 0.01039$	0.9965	0.01	0.03	81.2 ± 5.0
Sarafloxacin	$y = 0.01819x + 0.01140$	0.9994	0.02	0.07	81.6 ± 0.9
Sparfloxacin	$y = 0.06253x + 0.02989$	0.9969	0.02	0.06	73.6 ± 8.4
Flumequine	$y = 0.23182x + 0.16075$	0.9948	0.01	0.03	77.6 ± 4.5

Table S7. Summary of linearity, LODs, LOQs, and ME of 30 target analytes in shrimp samples

Compound	Regression equation (Internal)	R^2	LOD (μg/kg)	LOQ (μg/kg)	ME (%)
PFBS	$y = 0.59154x + 0.01513$	0.9995	0.01	0.03	59.1 ± 3.5
PFHxA	$y = 0.07395x + 0.00418$	0.9984	0.01	0.03	82.7 ± 2.8
HFPO-DA	$y = 0.04789x + 0.00327$	0.9991	0.02	0.07	70.1 ± 1.2
PFHpA	$y = 0.04623x + 0.00318$	0.9991	0.03	0.10	44.9 ± 7.0
PFHxS	$y = 0.31981x + 0.01095$	0.9994	0.03	0.10	154.2 ± 8.8
ADONA	$y = 0.28190x + 0.00354$	0.9998	0.03	0.10	46.4 ± 4.7
PFOA	$y = 0.31285x + 0.00973$	0.9976	0.02	0.07	77.4 ± 9.3
PFOS	$y = 0.58471x + 0.01577$	0.9995	0.03	0.10	90.5 ± 3.1
PFNA	$y = 0.05022x + 0.00410$	0.9989	0.02	0.07	106.5 ± 7.7
9Cl-PF3ONS	$y = 0.01616x + 0.04192$	0.9993	0.02	0.06	94.3 ± 4.9
PFDA	$y = 0.11397x + 0.01488$	0.9978	0.02	0.06	112.8 ± 0.2
NMeFOSAA	$y = 0.05512x + 0.00892$	0.9990	0.03	0.10	122.8 ± 3.0
PFUnA	$y = 0.08975x + 0.05217$	0.9922	0.07	0.23	137.8 ± 4.4
NEtFOSAA	$y = 0.03411x + 0.00839$	0.9929	0.19	0.63	129.2 ± 4.4
PFDoA	$y = 0.05940x + 0.03850$	0.9948	0.08	0.27	152 ± 4.6
PFTrDA	$y = 0.02764x - 0.00281$	0.9985	0.23	0.76	128.3 ± 2.9
PFTeDA	$y = 0.01869x + 0.00496$	0.9997	0.06	0.20	144 ± 2.0
Fleroxacin	$y = 0.02823x + 0.01461$	0.9980	0.12	0.40	71.6 ± 0.5
Ofloxacin	$y = 0.03315x + 0.02804$	0.9975	0.09	0.30	70.4 ± 0.9
Pefloxacin	$y = 0.06899x - 0.01585$	0.9968	0.11	0.37	79.0 ± 0.5
Enoxacin	$y = 0.05320x - 0.03552$	0.9925	0.04	0.13	98.8 ± 6.9
Enrofloxacin	$y = 0.01273x + 0.00220$	0.9975	0.16	0.53	80.2 ± 1.4
Danofloxacin	$y = 0.17646x - 0.06410$	0.9999	0.21	0.70	83.0 ± 7.3
Ciprofloxacin	$y = 0.03607x + 0.00747$	0.9966	0.02	0.07	90.8 ± 1.4
Orbifloxacin	$y = 0.15420x - 0.06691$	0.9910	0.14	0.47	69.9 ± 3.7
Lomefloxacin	$y = 0.05620x + 0.00955$	0.9996	0.01	0.03	75.6 ± 3.7
Difloxacin	$y = 0.11201x + 0.01039$	0.9965	0.01	0.03	76.4 ± 2.5
Sarafloxacin	$y = 0.01819x + 0.01140$	0.9994	0.02	0.07	80.2 ± 6.2
Sparfloxacin	$y = 0.06253x + 0.02989$	0.9969	0.03	0.10	71.4 ± 3.5
Flumequine	$y = 0.23182x + 0.16075$	0.9948	0.03	0.10	53.6 ± 0.7
